# The STEMI Mimic: Cocaine‐Associated Pulmonary Embolism With ST‐Segment Elevation

**DOI:** 10.1155/cric/9651125

**Published:** 2026-09-20

**Authors:** Kishan Reddy, Dhairya Nanavaty, Sohrab Singh, Parasuram Krishnamoorthy, Pedro Moreno

**Affiliations:** ^1^ St. John′s University, Jamaica, New York, USA; ^2^ The Mount Sinai Hospital, New York, New York, USA, mountsinai.org; ^3^ University of Oklahoma, Norman, Oklahoma, USA, ou.edu

## Abstract

Cocaine has been linked to both myocardial infarction (MI) and pulmonary embolism (PE), with the latter occasionally mimicking acute coronary syndrome. We report a 54‐year‐old man with polysubstance abuse who was found unresponsive and revived with naloxone. He developed chest pain with electrocardiogram showing ST‐segment elevation in V2–V3 and elevated troponin, suggestive of anteroseptal ST‐elevation MI (STEMI). Bedside echocardiography revealed right ventricular dilation and dysfunction without left ventricular abnormalities, prompting computed tomography angiography (CTA), which demonstrated large bilateral pulmonary emboli with right heart strain. He was anticoagulated and later discharged on a direct oral anticoagulant; coronary angiography revealed only mild nonobstructive disease. This case underscores the diagnostic challenge of distinguishing PE from STEMI in patients with cocaine use and highlights the utility of point‐of‐care echocardiography in avoiding unnecessary invasive procedures. Clinicians should consider PE in the differential diagnosis of chest pain with ST elevation.

## 1. Introduction

Cocaine has been implicated in the pathogenesis of both myocardial infarction (MI) and pulmonary embolism (PE) through several mechanisms, including endothelial injury, platelet activation, and systemic vasoconstriction [1–3] .

**Figure 1:**
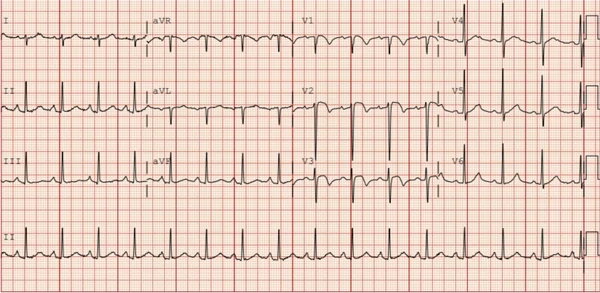
EKG on arrival for the patient suggestive of STE in V2–V3 with TWI.

**Figure 2:**
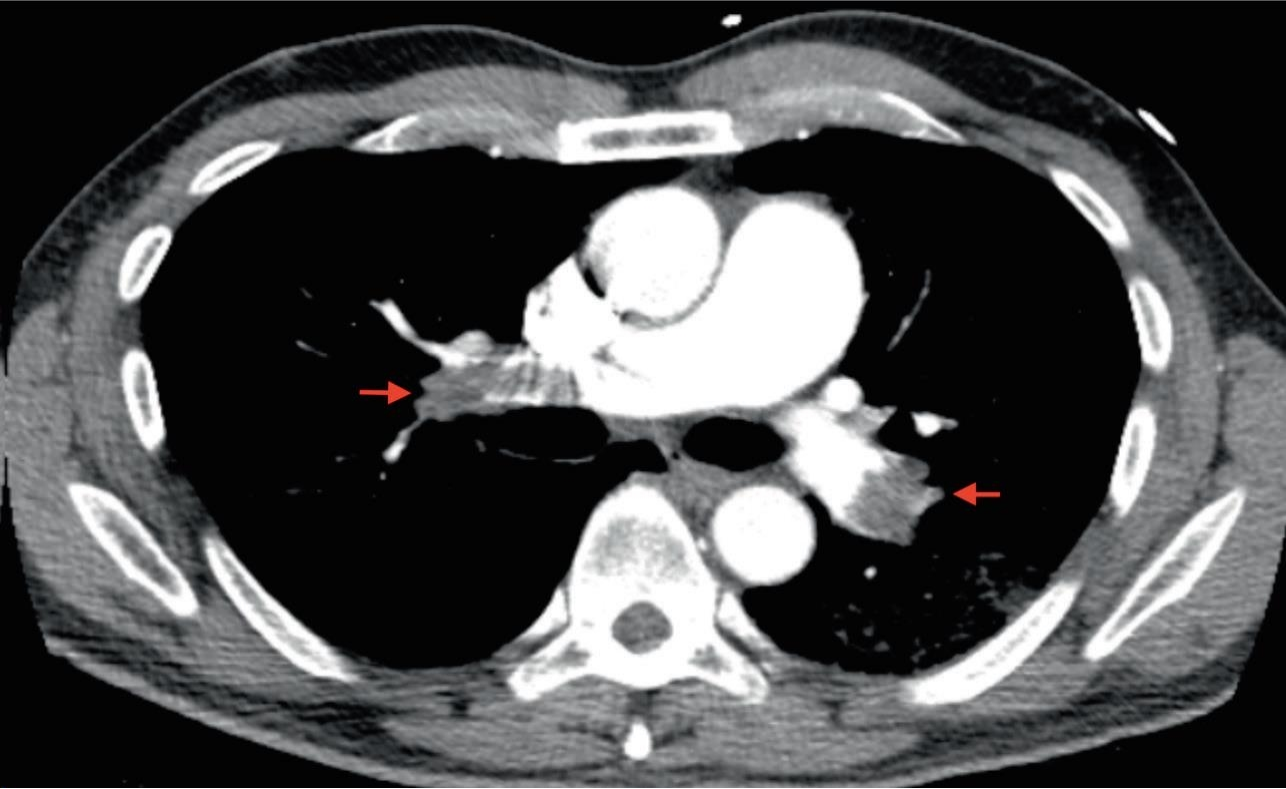
CT angiogram showing bilateral pulmonary embolism.

Although its association with MI is well documented, there is a growing body of case‐based evidence linking cocaine use to unprovoked PE, even in individuals without conventional thromboembolic risk factors [4–6].

Additionally, PE can clinically and electrocardiographically mimic MI, often presenting with ST‐segment elevations and elevated cardiac biomarkers, thereby leading to initial misdiagnosis and unnecessary invasive procedures such as coronary angiography. Several case reports have highlighted instances where patients with presumed acute coronary syndrome were ultimately diagnosed with PE after cardiac catheterization revealed no obstructive coronary disease [7–10].

We describe the case of a 54‐year‐old man with a history of polysubstance abuse who was found unresponsive and later presented with chest pain, ST‐segment elevation in leads V2–V3, and elevated troponin levels—findings highly suggestive of anteroseptal ST‐elevation MI (STEMI). Further evaluation revealed large bilateral pulmonary emboli with right ventricular strain, underscoring the diagnostic complexity in cases where cocaine use, EKG abnormalities, and myocardial injury biomarkers overlap.

## 2. Case Report

A 54‐year‐old male with a medical history of bipolar disorder and known polysubstance abuse was found unresponsive on the street by emergency medical services. He regained consciousness following administration of intranasal naloxone. Upon awakening, the patient complained of severe, central chest pain. Aspirin 324 mg was administered, and the patient was brought into the Emergency Department (ED).

Upon arrival at the ED, vital signs were notable for tachycardia but no hypotension or hypoxia. The initial EKG showed sinus rhythm with 1–2 mm ST‐segment elevation in leads V2–V3 and T wave inversions (Figure 1), raising concern for anteroseptal STEMI. Laboratory workup revealed a high‐sensitivity troponin of 435 ng/L, BNP of 170 pg/mL, WBC count of 16,000/*μ*L, and elevated inflammatory markers. A urine toxicology screen was positive for methadone and cocaine.

Given the concerning EKG and elevated troponin, the interventional cardiology team was consulted for emergent catheterization. However, point‐of‐care transthoracic echocardiography demonstrated a normal left ventricular ejection fraction, no regional wall motion abnormalities, and a moderately dilated right ventricle with depressed systolic function (Video S1). Based on these findings, the cardiac catheterization was deferred, and a computed tomography angiography (CTA) was performed.

Imaging revealed large, bilateral acute pulmonary emboli with signs of RV strain, along with background bullous emphysematous changes (Figure 2). A continuous intravenous heparin infusion was initiated. Interventional radiology was consulted, and after a multidisciplinary discussion, the consensus was to proceed with medical management alone, given the patient′s hemodynamic stability. Of note, the patient had a duplex ultrasound scan of the lower extremities performed, which was not suggestive of any deep vein thrombosis and was essentially a normal study.

Over the following 24 h, troponin levels trended down to 349 ng/L. Despite clear evidence pointing to PE as the etiology of his symptoms, the patient remained concerned about possible coronary artery disease (CAD) because of a strong family history. After shared decision‐making and a thorough risk‐benefit discussion, a diagnostic coronary angiogram was performed, which revealed mild, nonobstructive CAD.

The patient was discharged in a stable condition on a direct oral anticoagulant (DOAC) and referred for outpatient hematology‐oncology follow‐up for evaluation of potential hypercoagulable states and malignancy screening, given the unprovoked nature of the PE.

## 3. Discussion

Our case represents a rare but important scenario in which PE masquerades as an anteroseptal MI. A varying percentage of patients with chest pain and prehospital ST elevation have diagnoses other than acute MI (AMI) [11, 12]. Differentiating ST elevation is challenging, with etiologies including but not limited to MI, pericarditis, PE, electrolyte imbalances, other gastrointestinal, CNS, and pulmonary causes, [13–15].

The overall incidence rate of ST elevation in patients presenting with acute PE is unknown. Patients diagnosed with PE presenting with symptoms such as chest pain, dyspnea, or syncope, alongside characteristic electrocardiographic findings including right bundle branch block (RBBB) and ST‐segment elevation mainly in the anterior leads (V1–V4), have been described. Zelfani et al. and Siddiqa et al. reported similar cases in which patients with PE had anteroseptal ST elevations, leading to misdiagnosis of STEMI [16, 17]. Bruss et al. reported a couple of cases in which one patient had ST elevation in V1 and V2, whereas the other had ST elevation in aVR and V1 with diffuse ST depression in other leads; both ultimately diagnosed with PE [18]. Ghonem et. al described a case in which a saddle PE manifested as ST elevations in V1–V4 and deep T wave inversions in inferior leads. They also demonstrate how bedside ultrasound was instrumental in making the diagnosis. [19] Tomaz et al. described a 57‐year‐old male with PE who exhibited ST elevation in V1–V4 and T wave inversion in lead III with angiography revealing occlusion of the conus artery due to paradoxical embolism [20]. Wilson et al. reported a patient presenting with syncope with EKG suggestive of RBBB, ST elevations, and Q waves found to have mild to moderate coronary atherosclerosis on angiographic evaluation [8]. Lin et al. detailed a case with incomplete RBBB and ST elevation with normal coronary arteries [21]. Falterman et al. documented a case of syncope with sudden hemodynamic decompensation leading to an unfortunate mortality. EKG was suggestive of ST elevation in V1–V4. Autopsy revealed PE without coronary obstruction [22]. Haghi et al. described paradoxical embolism causing non‐ST elevation MI with obstructive left marginal artery in a patient with PE [23].

As seen from the cases above, ST‐segment elevation in the anteroseptal leads is an uncommon but recognized finding in acute PE. The pathophysiology remains unclear, but several mechanisms have been proposed: right ventricular strain from sudden pressure overload causing myocardial ischemia; hypoxemia‐induced catecholamine surge increasing myocardial oxygen demand; and paradoxical embolism through a patent foramen ovale causing coronary occlusion. [21, 23]

Cocaine use is increasingly recognized as a contributor to PE through endothelial injury, platelet activation, and systemic hypercoagulability, fulfilling Virchow′s triad [24–26]. Prior cases report cocaine abuse as a potential causative factor for PE [5, 27, 28]. Our case showed a similar association of PE in a polysubstance abuser positive for cocaine and methadone.

This case highlights several key teaching points. First, it adds to the growing evidence suggesting an association between cocaine use and increased risk of PE, though the exact etiology remains uncertain. Second, it reinforces that PE, though rare, can mimic ST‐elevation MI both electrocardiographically and biochemically. Third, bedside point‐of‐care ultrasound plays a crucial role in rapidly identifying life‐threatening conditions such as right heart strain from massive PE, significantly influencing management decisions. Maintaining a broad differential and integrating clinical, laboratory, and imaging data are essential to avoid misdiagnosis and optimize patient care.

## Funding

No funding was received for this manuscript.

## Ethics Statement

Ethical approval was not required for this case report in accordance with local institutional policies.

## Consent

Written informed consent was obtained from the patient for publication of this case report and accompanying images.

## Conflicts of Interest

The authors declare no conflicts of interest.

## Supporting information


**Supporting Information** Additional supporting information can be found online in the Supporting Information section. Video S1: Echocardiographic evidence showing dilation and decreased function of the right ventricle (RV), demonstrating RV strain.

## Data Availability

Data sharing is not applicable to this article as no datasets were generated or analyzed during the current study.
